# Preparation of Polyethylene/α-Zirconium Phosphate Nanocomposites via a Well-Controlled Polyethylene-Grafted Interface

**DOI:** 10.1021/acs.langmuir.3c00058

**Published:** 2023-04-13

**Authors:** Mingzhen Zhao, Hong-Mao Wu, Hengxi Chen, Guan-Hui Lai, Zewen Zhu, Jen-Long Wu, Wen-Hao Kang, Hung-Jue Sue

**Affiliations:** † Department of Material Science and Engineering, Texas A&M University, College Station, Texas 77843, United States; ‡ Polyolefin Department of Formosa Plastics Corporation, Yunlin County 63801, Taiwan

## Abstract

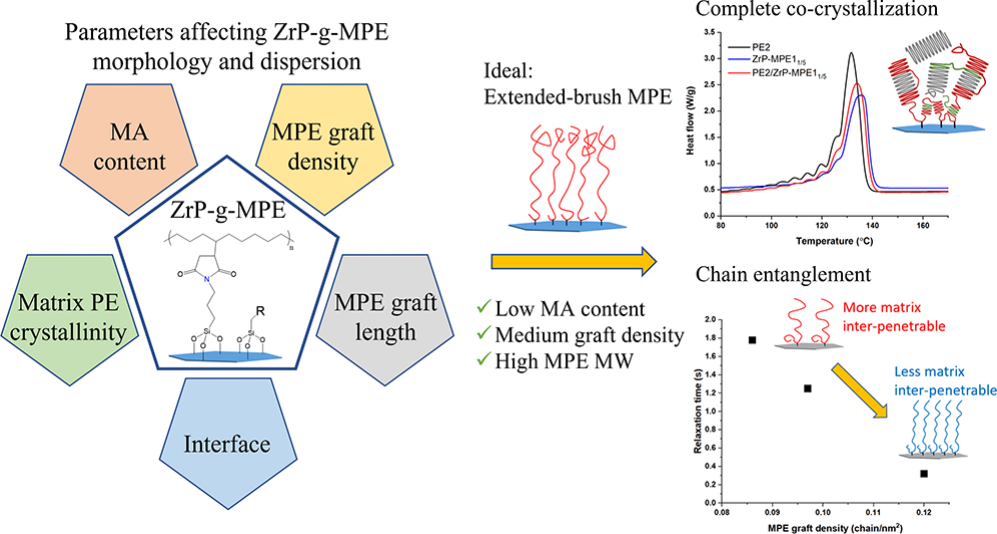

It is a daunting task to prepare polyolefin nanocomposites that contain well-exfoliated nanoplatelets due to the nonpolar and high crystallinity nature of polyolefins. In this research, a robust approach was developed to prepare polyethylene (PE) nanocomposites by grafting maleated polyethylene (MPE) onto pre-exfoliated α-zirconium phosphate (ZrP) nanoplatelets via a simple amine-anhydride reaction to form ZrP-*g*-MPE. Several variables, including maleic anhydride (MA) content, MPE graft density, MPE molecular weight, and PE matrix crystallinity, were investigated to determine how they influence ZrP-*g*-MPE dispersion in PE. It was found that grafted PE has a different morphology and that the long PE brushes with medium graft density on ZrP can achieve sufficient chain entanglement and cocrystallization with PE matrix to stabilize and maintain ZrP-*g*-MPE dispersion after solution or melt mixing. This leads to enhanced Young’s modulus, yield stress, and ductility. The structure–property relationship of PE/ZrP-*g*-MPE nanocomposites and usefulness of this study for the preparation of high-performance polyolefin nanocomposites are discussed.

## 1 Introduction

Polymer nanocomposites containing well-exfoliated nanoplatelets can lead to significant improvement in mechanical, electrical, thermal, dielectric, and optical properties.^1−8^ The polymer–nanoparticle interface plays an important role in determining nanoparticle dispersion and property enhancement.^1,9−11^ Polymer grafting onto nanoparticle surfaces has been demonstrated as one of the ways to modify the polymer–nanoparticle interface and improve compatibility. Polymer graft density and its chain length are among the key parameters for controlling interfacial interaction.^12^

Polymer grafting onto a substrate surface may result in a mushroom-like or extended polymer brush-like conformational assembly, depending on the substrate grafting density and brush length.^13^ The loosely grafted chains tend to form a mushroom-like morphology, and the densely grafted scenario shows an extended polymer brush-like assembly. Different polymer grafting morphologies on nanoparticle surfaces result in different polymer–nanoparticle interfacial characteristics. It has been demonstrated that the chain length and graft density of semidilute polymer brush-like poly(methyl methacrylate) (PMMA) grafted ZrP affect chain entanglement and ZrP dispersion after mixing with the PMMA matrix.^9^ In the ZrP-*g*-PMMA system with medium graft density and long graft length, concentrated polymer brushes and semidilute polymer brushes coexist to induce sufficient chain entanglement, which stabilizes ZrP dispersion. Because of the semicrystalline nature of polyethylene (PE), the crystallization process also plays an important role in affecting nanoparticle dispersion.^14^ It has been reported that the dispersion of SiO_2_-*g*-PE nanoparticles in PE depends on PE graft density, which affects crystallization kinetics and intermolecular penetration with the matrix.^14^ It was also reported that polyethylene oxide (PEO) absorbed on a silica surface was able to cocrystallize with the PEO matrix. Cocrystallization at the interface forms a strong bonding between nanoparticles and the polymer matrix.

Thermal fractionation is one way to characterize phase separation and cocrystallization behaviors in semicrystalline polymers.^15−17^ In short, polymer crystallization processes are classified into three regimes. Regime I represents the fully melted scenario with no nucleation occurring at the temperature above melting. In Regime II, self-nucleation takes place without annealing of polymer crystals. Self-nucleation of molten polymers and annealing of unmolten crystals coexist in Regime III. The transition temperature between Regimes II and III is the ideal self-nucleation temperature (*T*_s_). Successive self-nucleation/annealing (SSA) was performed starting from the assigned *T*_s_. The final heating profile reflects the polymer crystallization information obtained via the controlled SSA procedures. Cocrystallization of LLDPE/HDPE^18^ or LLDPE/wax^16^ blends has been characterized by using SSA. This technique is applied in the present study to characterize the cocrystallization phenomenon between ZrP-*g*-MPE and matrix PE.

As shown in our earlier work,^1,19^ MPE can be covalently grafted on exfoliated ZrP surfaces to achieve controlled graft density via a simple amine-anhydride reaction. By controlling MPE graft density, ZrP-*g*-MPE may remain well-exfoliated in the PE matrix. Since MA groups are distributed along the PE backbone, MPE grafting on ZrP is not a chain-end grafting morphology.^20^ Depending on the MA content, a long-chain polymer brush or mushroom-like morphology may be formed, which affects the PE and ZrP-*g*-MPE interface and ZrP exfoliation in the PE matrix.

In this work, commercial MPE with different MA contents and molecular weights were grafted onto exfoliated ZrP surfaces. The grafted MPE morphology and crystallinity of the matrix PE are found to play important roles in ZrP exfoliation. The effect of chain entanglement and cocrystallization between grafted MPE and matrix PE on ZrP exfoliation was systematically investigated to establish their structure–property relationship. The implication of the above findings on improving PE properties is discussed.

## 2 Experimental Section

### 2.1 Materials

Zirconyl chloride (ZrOCl_2_·8H_2_O, 98%, Sigma-Aldrich), phosphoric acid (85%, EM Science), and all of the solvents are reagent grade and used as received. Jeffamine M1000 with a molecular weight of 1000 g/mol was donated by the Huntsman Corporation. 3-Aminopropyl trimethoxysilane (≥97%) was purchased from Alfa Aesar. Octadecyl trimethoxysilane (ODMS) (≥90%) was purchased from Gelest. MPE and PE used in this study are listed in Table 1. PE1 has a more uniform structure with narrower molecular weight distribution compared to PE2. As a result, PE1 is a simpler matrix to study the PE/ZrP interfacial characteristics and their mechanical property relationship.

**Table 1:** MPE and PE Information

name		MA content (%)	*T*_m_ (°C)	*M*_W_ (g/mol)	MWD	supplier
MPE	MPE1	0.2	133	130,600	5.32	Dow (BYNEL 40E529)
	MPE2	1.4	131	159,000	3.30	BYK (SCONA TPPE 1212)
	MPE3	1.5	127	49,900	4.07	BYK (SCONA TSPE 2102)
neat PE	PE1		134	75,000	5	Formosa Plastics (HDPE 8050)
	PE2		131	140,700	21	Formosa Plastics (HDPE 9007)

### 2.2 Preparation of PE/ZrP-*g*-MPE Nanocomposites

#### 2.2.1 Grafting of MPE to Exfoliated ZrP Surface (ZrP-*g*-MPE)

ZrP synthesis, exfoliation, and functionalization were performed using a method we have previously described.^11^ In brief, ZrP with a lateral dimension of 100 nm was synthesized via a reflux method. Then, ZrP was dispersed in acetone and exfoliated by reacting with M1000 (ZrP-M1000). The ZrP-M1000 was transferred to xylene and reacted with APTMS (APTMS is 5 mol % to ZrP) at 70 °C for 24 h in nitrogen. The ZrP-M1000-APTMS was then used for MPE grafting. To use the ZrP-*g*-MPE1_1/5_ as an example, 2 g of MPE1 was dissolved in 100 mL of xylene at 110 °C. ZrP-M1000-APTMS (0.31 g) in 10 mL of xylene solution was added dropwise into the stirring MPE1 solution. The MPE1/ZrP mixture was reacted for 24 h in a nitrogen atmosphere. Then, 7.75 g of octadecyl trimethoxysilane (ODMS) was added and reacted for 24 h to remove M1000 and reduce surface polarity. After the reaction, the solution was cooled to room temperature, followed by precipitation in an excess amount of tetrahydrofuran and centrifuged at 8000 rpm. The collected precipitates were rinsed with tetrahydrofuran and centrifuged three times to remove any unreacted reactants.

#### 2.2.2 Solution Mixing of Neat PE and ZrP-*g*-MPE

Neat PE and ZrP-*g*-MPE were separately dissolved in xylene at 120 °C. Then, the neat PE was transferred to the stirring ZrP-*g*-MPE solution. The mixed solution was stirred for 1 h in nitrogen. The final solution-mixed PE/ZrP was cooled down and precipitated in excess methanol and centrifuged to collect the sediments. PE/ZrP was dried at 120 °C in an oven for 24 h to remove the remaining solvent.

#### 2.2.3 Melt-Mixed Neat PE with ZrP-*g*-MPE

ZrP-*g*-MPE1_1/5_ (ZrP 0.8 g) was first solution-mixed with 10 g of neat PE in xylene at 120 °C for 1 h to prepare the PE1/ZrP-*g*-MPE1_1/5_ masterbatch. Then, the masterbatch was dried overnight to remove the solvent. The PE/ZrP masterbatch was mixed with 30 g of neat PE1 and transferred into a 60 mL HAAKE mixer at 180 °C with a rolling speed of 50 rpm/min for 7 min.

### 2.3 Characterization

#### 2.3.1 Thermogravimetric Analysis (TGA)

TGA measurements were carried out using a TA Instruments Q500 thermogravimetric analyzer. The temperature was increased from 30 to 800 °C at 20 °C/min under 60 mL/min nitrogen flow. TGA was used to determine the organic component of modified ZrP and ZrP concentration in the nanocomposites.

#### 2.3.2 Differential Scanning Calorimetry (DSC)

DSC was performed using TA Q20 with a nitrogen flow. Crystallinity was calculated by , where Δ*H*_f_ is the heat fusion obtained from calorimetry, *m* is the specimen weight, and Δ*H*_f_^0^ is the enthalpy of 100% crystalline polyethylene, which is 293 J/g.

#### 2.3.3 Wide Angle X-Ray Diffraction (WAXS)

The WAXS pattern was obtained using a Bruker D8 focus Bragg-Brentano X-ray powder diffractometer, with Cu Kα radiation (λ = 1.54178 Å).

#### 2.3.4 Small Angle X-Ray Scattering (SAXS)

SAXS was measured using a Rigaku-3000 instrument. Characteristic Cu X-rays with a wavelength (λ) of 1.542 Å were generated using a rotating copper anode (MicroMax-007HFM, Rigaku).

#### 2.3.5 Transmission Electron Microscopy (TEM)

TEM images were obtained from a JEOL JEM-1200. Ultrathin sections with thicknesses of 80–100 nm were prepared using a Reichert-Jung Ultracut E microtome with a diamond knife at cryogenic temperature.

#### 2.3.6 Rheology

Rheological behavior was analyzed with an ARES-G2 (TA Instruments), using 8 mm aluminum parallel plates. The gap between the two plates was set between 0.7 and 1.5 mm. Rheological measurements were performed at 160 °C with a strain amplitude of 1.0% and angular frequency from 0.01 to 100.0 rad/s.

#### 2.3.7 Tensile Test

The uniaxial tensile tests were conducted on Type-V specimens (ASTM D638) at a crosshead speed of 10 mm/min. Four measurements were conducted to obtain the average tensile property.

## 3 Results and Discussion

### 3.1 ZrP Exfoliation in PE

**Figure 1:**
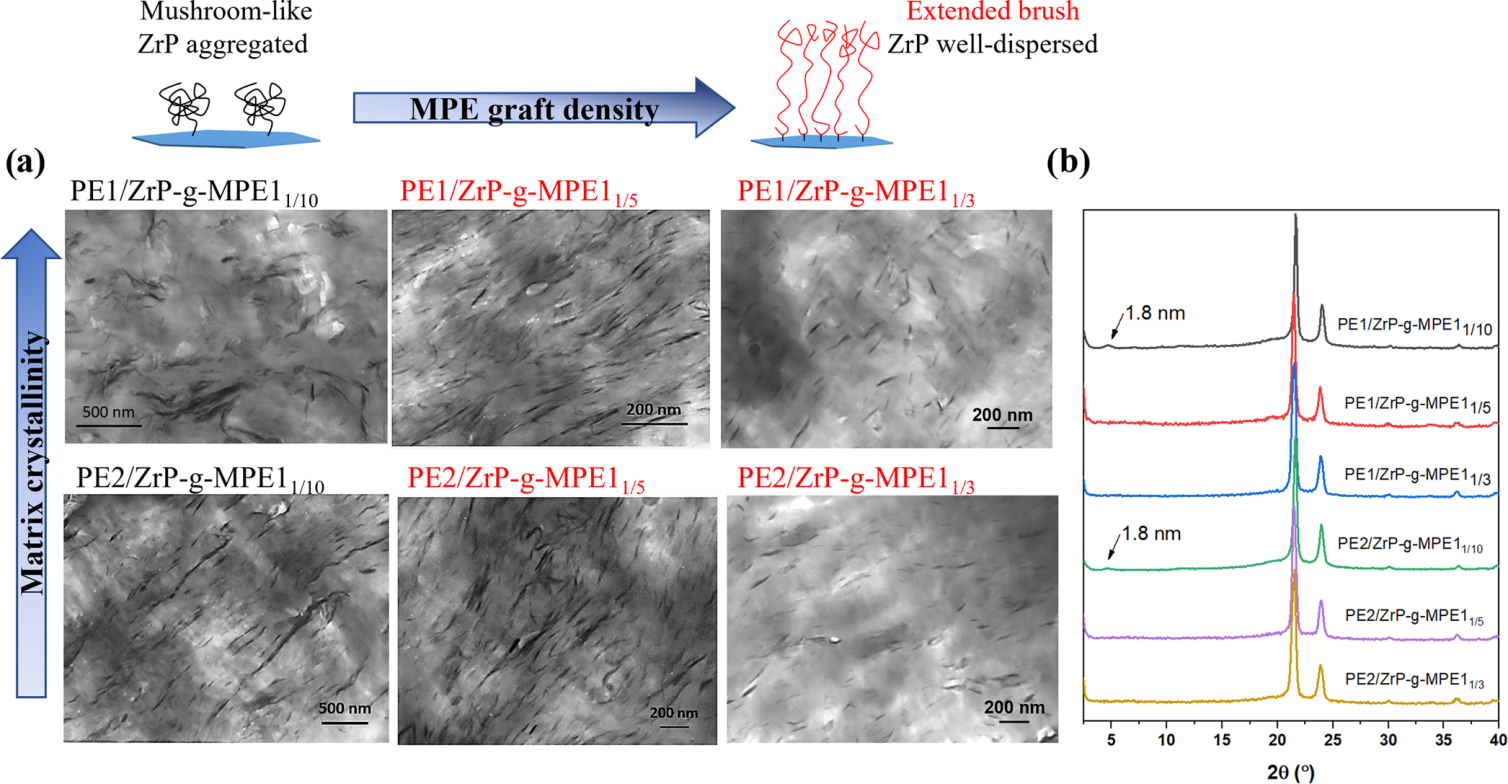
(a) TEM of PE1/ZrP-*g*-MPE1 and PE2/ZrP-*g*-MPE1 with different MPE1 graft densities. (b) WAXS of PE1/ZrP-*g*-MPE1 and PE2/ZrP-*g*-MPE1.

MPE was successfully grafted onto the exfoliated ZrP surface via an amine-anhydride reaction.^11^ Three different types of MPE were selected (Table 1). The MA groups are assumed to distribute uniformly on the PE backbone. The number of reactive sites on MPE is determined by the MA content, which affects the MPE grafting morphology on ZrP. The MPE graft density on ZrP can be controlled by adjusting the molar ratio of MA on MPE and NH_2_ on ZrP. The MPE graft density of the designed ZrP-*g*-MPE model systems is listed in Table 2. At a similar molar ratio of MA/NH_2_, MPE1 graft density is higher than MPE2 and MPE3. Grafted MPE1 with a lower MA content tends to graft more efficiently on the ZrP surface and results in a more spread-out long-chain brush morphology.^13^ With low PE graft density, mushroom-like morphology is formed and is unable to provide sufficient coverage to ZrP. At an appropriate PE graft density, MPE1 forms a more extended brush-like morphology and is able to entangle and cocrystallize with the matrix PE. However, MPE with a higher MA content has a higher number of reactive sites on the PE backbone, which formed a mushroom-like shorter chain morphology. This mushroom-like MPE grafting may cause gelling and be ineffective to form entanglement with matrix PE. Our earlier results show that grafting MPE3 onto an already exfoliated ZrP causes gelling at a graft density lower than 0.087 chain/nm^2^.^11^ An ideal grafted MPE structure is the semidilute polymer brush chain morphology, which minimizes gelling and also stabilizes ZrP exfoliation (Figure 1).^9^

**Table 2:** Designed ZrP-*g*-MPE Model Systems and Their Corresponding MPE Graft Density on ZrP

name	molar ratio MA/NH_2_	ZrP (wt %)	ZrP after extraction (wt %)	graft density (chain/nm^2^)	abbrev. notation
ZrP-*g*-MPE1	1/10	20	40	0.020	ZrP-*g*-MPE1_1/10_
	1/5	13	16.7	0.086	ZrP-*g*-MPE1_1/5_
	1/4	11	14.6	0.097	ZrP-*g*-MPE1_1/4_
	1/3	6.8	12.7	0.120	ZrP-*g*-MPE1_1/3_
	1/1	2.8	6.2	0.430	ZrP-*g*-MPE1_1/1_
ZrP-*g*-MPE2	1/2	19.5	43.3	0.007	ZrP-*g*-MPE2_1/2_
	1/1	14.3	40.5	0.009	ZrP-*g*-MPE2_1/1_
	2/1	9.3	33	0.012	ZrP-*g*-MPE2_2/1_
ZrP-*g*-MPE3	1/1	15	40	0.010	ZrP-*g*-MPE3_1/1_
	2/1	10	25	0.087	ZrP-*g*-MPE3_2/1_
	5/1	6	19	0.140	ZrP-*g*-MPE3_5/1_

ZrP-*g*-MPE1 is an ideal model system for the structure–property relationship studies. ZrP with four different MPE1 graft densities were designed to investigate the graft density effect on the PE–ZrP interface and how the interfacial characteristics affect ZrP dispersion in PE. ZrP-*g*-MPE1_1/10_ has a graft density of 0.02 chain/nm^2^. The completely dried ZrP-*g*-MPE1_1/10_ can still be fully redissolved in hot xylene. Figure 1 shows the TEM and WAXS of ZrP-*g*-MPE1 dispersion in high- (PE1) and low- (PE2) crystallinity PE matrices. All PE/ZrP-*g*-MPE1 nanocomposites contain 3 wt % of inorganic ZrP. ZrP-*g*-MPE1_1/10_ with the lowest MPE1 graft density forms an aggregated structure in both PE1 and PE2. Microscale ZrP clusters and a trace amount of ZrP intercalation of 1.8 nm were observed using WAXS. Too low a graft density does not generate sufficient grafted PE covering and results in ZrP aggregation and possible restacking in PE. On increasing the MPE1 graft density to 0.086 and 0.12 chain/nm^2^, grafted MPE provides sufficient coverage and strong interface with matrix PE, and ZrP-*g*-MPE1_1/5_ remains well dispersed in both PE1 and PE2.

Polymer-grafted nanoparticle dispersion in a polymer matrix is affected by the polymer graft density (**σ**) and the ratio between the molecular weight of the matrix polymer (**P**) and grafted (**N**) polymer chains.^21,22^ When **σ** is below the allophobic limit (σ*),^21,22^ grafted polymer is insufficient to cover nanoparticle surfaces, which results in nanoparticle aggregation. As **σ** increases above σ^*^, grafted and matrix polymer chains can interpenetrate and may lead to repulsive interaction between nanoparticles to achieve good dispersion. With further increased **σ** or **P/N** ratio, the grafted polymer chains become too crowded to allow for matrix molecular interpenetration to form strong entanglement, which results in attractive interaction and particle aggregation (autophobic phase transition, σ^**^). In this PE/ZrP-*g*-MPE1 model system, the allophobic limit is 0.02 < σ^*^ < 0.086 chain/nm^2^ in both PE1 and PE2 matrices. The autophobic limit of the ZrP-*g*-MPE1 model system should be higher than 0.43 chain/nm^2^. Any ZrP-*g*-MPE1 with MPE1 graft density in between allophobic and autophobic limits should remain well dispersed in PE.

**Figure 2:**
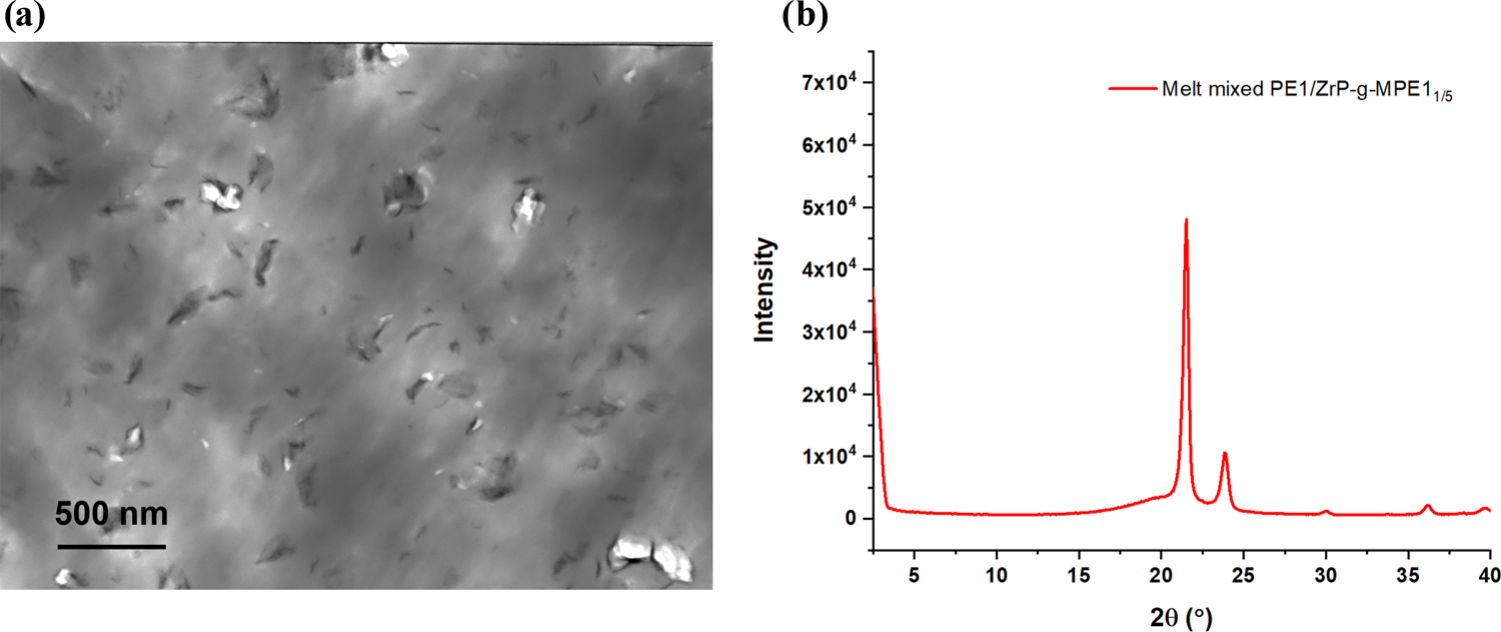
(a) TEM and (b) WAXS of melt-mixed PE/ZrP-*g*-MPE1_1/5_ containing 2 wt % of ZrP.

It is well known that melt dispersion of nanoparticles in a polymer matrix has a much higher entropic penalty to overcome compared to solution mixing.^23^ It is still a major challenge to achieve good dispersion of two-dimensional (2D) nanoplatelets in polyolefin matrices through melt mixing. To show the potential usefulness of the present study, the ZrP-*g*-MPE1_1/5_ was melt-mixed with PE1 to determine if polyolefin nanocomposites can be melt-processed. As shown in Figure 2a, ZrP-*g*-MPE1_1/5_ is found to be well dispersed throughout the entire PE1 matrix; no detectable ZrP stacking could be observed via WAXS (Figure 2b). The strong interfacial entanglement and cocrystallization between PE1 and ZrP-*g*-MPE1_1/5_ can overcome the entropy penalty of melt mixing and stabilizes dispersion of ZrP-*g*-MPE1_1/5_ in PE1. Investigation of the effect of PE–ZrP interfacial interaction on ZrP dispersion is discussed further in Section 3.2.

**Figure 3:**
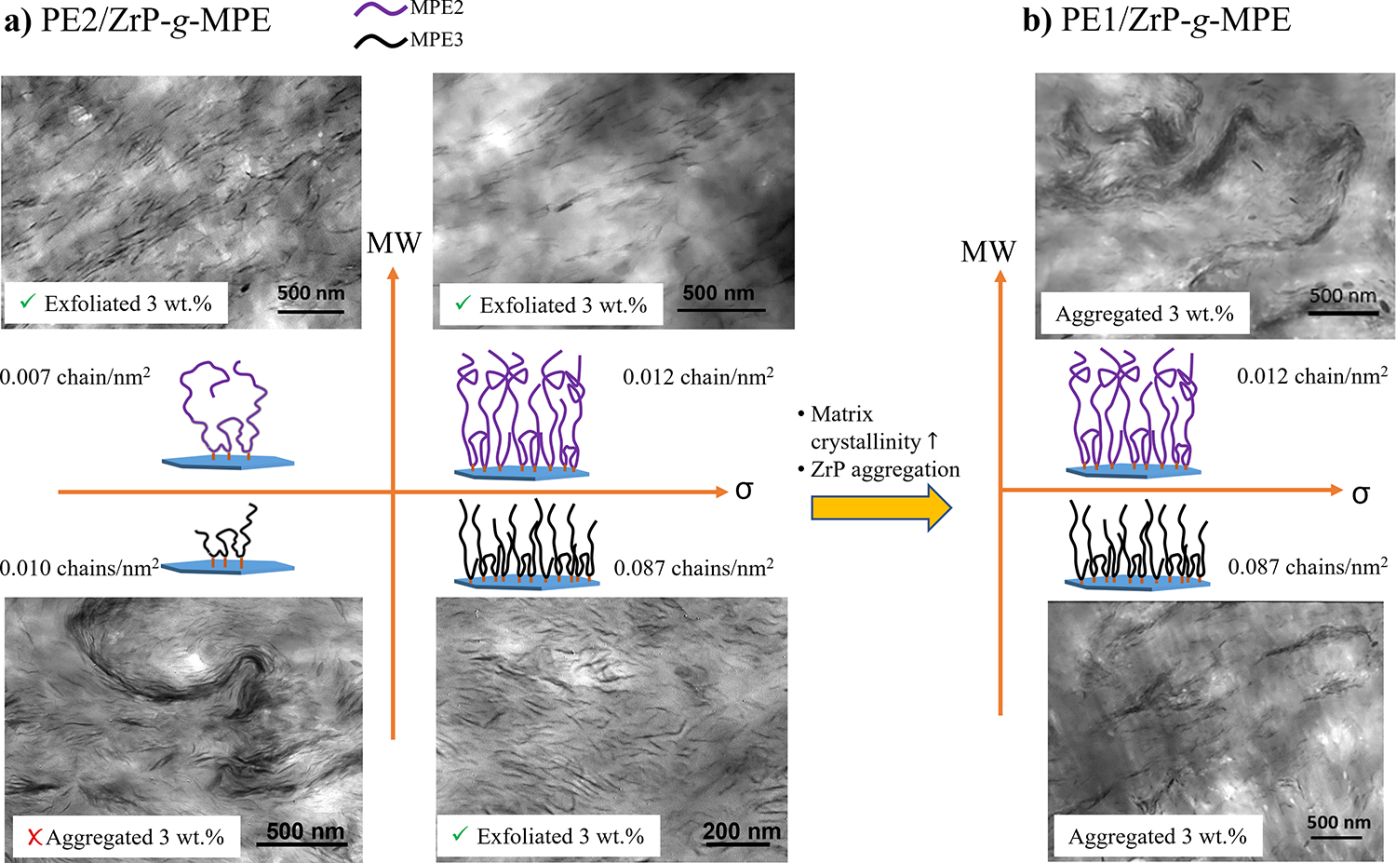
TEM of (a) PE2/ZrP-*g*-MPE2 and PE2/ZrP-*g*-MPE3 and (b) PE1/ZrP-*g*-MPE2_2/1_ and PE1/ZrP-*g*-MPE3_2/1_ with different graft densities.

Compared to MPE1, MPE2 and MPE3 contain much higher MA content. The higher number of reactive sites on the MPE backbone results in a mushroom-like chain morphology on the ZrP surface. MPE2 and MPE3 have different molecular weights (MW), which were chosen to investigate the effect of MPE MW on ZrP dispersion in PE. As shown in Figure 3, ZrP-*g*-MPE2_1/2_ can remain well dispersed in the PE2 matrix when the graft density is higher than 0.007 chain/nm^2^. However, ZrP-*g*-MPE3_1/1_ with a similar graft density (0.010 chain/nm^2^) to ZrP-*g*-MPE2_1/2_ forms an aggregated ZrP structure in PE2. ZrP-*g*-MPE3 is well dispersed in PE2 when a graft density is higher than 0.087 chain/nm^2^. MPE2 with a higher MW compared to MPE3 can better stabilize ZrP dispersion at a lower graft density in PE2. However, ZrP-*g*-MPE2_2/1_ and ZrP-*g*-MPE3_2/1_ cannot achieve good dispersion in high crystallinity PE1 matrix. The significant differences in ZrP dispersion among ZrP-*g*-MPE1, ZrP-*g*-MPE2, and ZrP-*g*-MPE3 in the PE matrix are due to the different ZrP-*g*-MPE morphologies at the PE–ZrP interface.

### 3.2 PE–ZrP Interfacial Characterization

Possible cocrystallization between ZrP-*g*-MPE and PE systems was investigated. PE exhibits high crystallinity with a fast crystallization rate, which may affect nanoparticle dispersion upon cooling.^14^ In the molten state, chain entanglement between ZrP-*g*-MPE and matrix PE determines the stability of ZrP dispersion. In the solid state, PE matrix cocrystallization with ZrP-*g*-MPE ensures good interfacial bonding and dispersion of ZrP.

A thermal fractionation successive self-nucleation/annealing (SSA) technique was employed to investigate cocrystallization between grafted MPE on ZrP and PE matrix.^24^ Before conducting SSA, an ideal self-nucleation temperature (*T*_s_) was identified (Table 3). SSA was applied to PE1, PE2, ZrP-*g*-MPE1_1/5_, ZrP-*g*-MPE2_2/1_, and ZrP-*g*-MPE3_2/1_. The designated *T*_s_ for each PE/ZrP-*g*-MPE system is listed in Table 4. The same SSA procedure was applied to each system for comparison. Using PE1/ZrP-*g*-MPE1_1/5_ as an example, *T*_s_ of PE1 and ZrP-*g*-MPE1_1/5_ are 128.5 and 129 °C, respectively. To ensure no annealing of each component during the 1^st^ SSA isothermal cycle, the highest *T*_s_ should be selected. Isothermal crystallization and annealing were conducted at 5 °C per step from 129 °C down to 59 °C for all of the samples. The final heating profile is used for determining their possible cocrystallization behavior.

**Table 3:** Ideal Self-Nucleation Temperature for Neat PE and ZrP-*g*-MPEs

	PE1	PE2	ZrP-*g*-MPE1_1/5_	ZrP-*g*-MPE2_2/1_	ZrP-*g*-MPE3_2/1_
*T*_s_ (°C)	128.5	128	129	127.5	125.5

**Table 4:** Designated Self-Nucleation Temperature of PE/ZrP-*g*-MPEs for SSA Profile

*T*_s_ (°C) of PE/ZrP-*g*-MPE	ZrP-*g*-MPE1_1/5_	ZrP-*g*-MPE2_2/1_	ZrP-*g*-MPE3_2/1_
PE1	129	128.5	128.5
PE2	129	128	128

**Figure 4:**
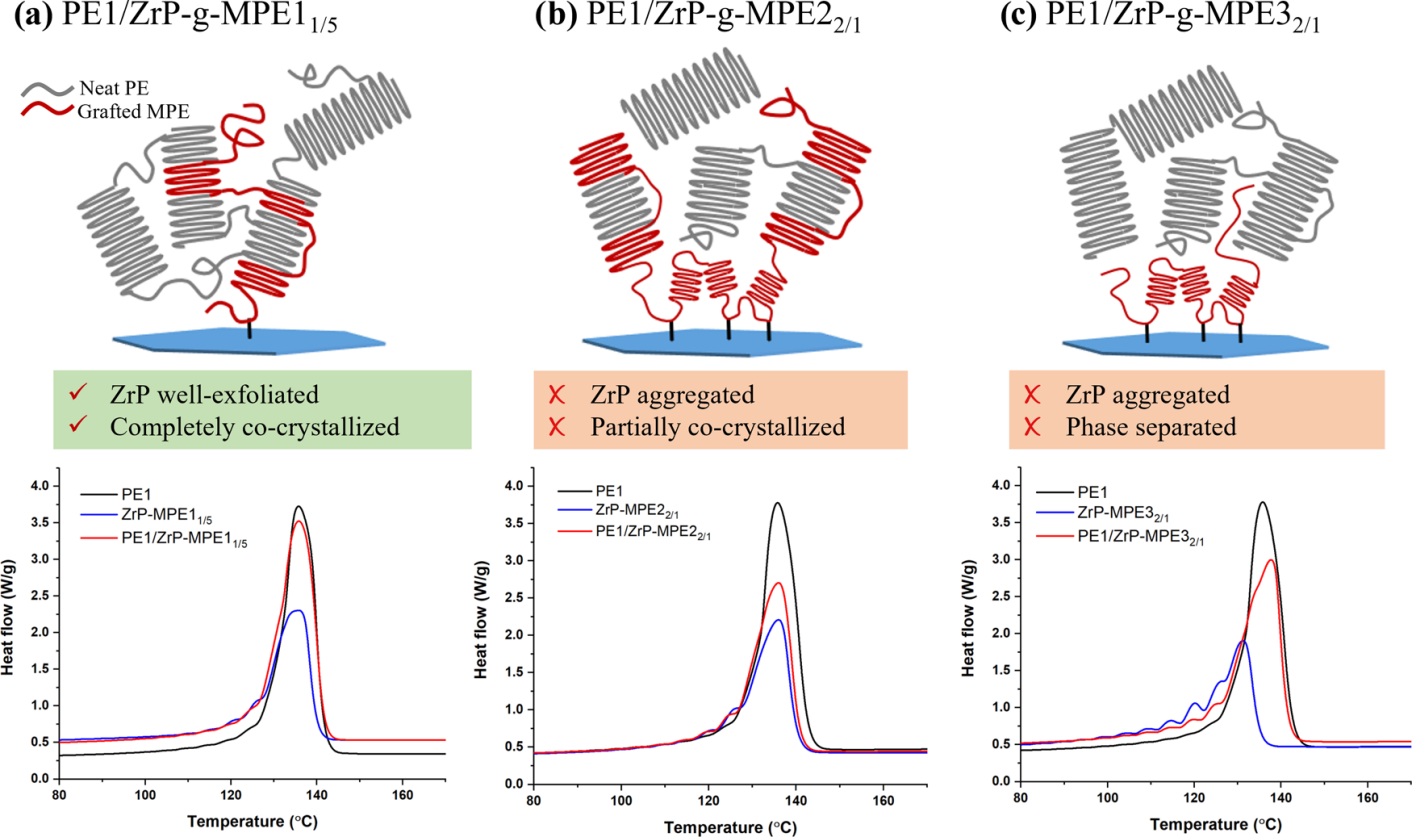
SSA profiles of (a) PE1, ZrP-*g*-MPE1_1/5_ and PE1/ZrP-*g*-MPE1_1/5_, (b) PE1, ZrP-*g*-MPE2_2/1_ and PE1/ZrP-*g*-MPE2_2/1_, and (c) PE1, ZrP-*g*-MPE3_2/1_, and PE1/ZrP-*g*-MPE3_2/1_.

Figure 4 shows the SSA results and their schematic interfacial morphology. It is important to note that the first isothermal step in SSA corresponds to the ideal self-nucleation; no crystal fractionation occurs. The temperature at which the first fractionation occurs is 5 °C lower than the ideal *T*_s_. Each melting peak corresponds to a specific thermal fractionation step. The first five fractionation temperatures and their corresponding melting temperatures are listed in Table 5. The overlap of *T*_m_ peaks or peak shifts are evidence for cocrystallization between polymers.^18^ PE1, with a narrower MWD, possesses only a main melting peak. In Figure 4a, PE1, ZrP-*g*-MPE1_1/5_, and PE1/ZrP-*g*-MPE1_1/5_ all exhibit a similar melting behavior. Melting temperatures of PE1/ZrP-*g*-MPE1_1/5_ are located in between PE1 and ZrP-*g*-MPE1_1/5_, which indicates a complete cocrystallization at the interface, which stabilizes ZrP-*g*-MPE dispersion in PE matrix. This finding also corroborates with the TEM and WAXS findings shown in Figure 1.

**Table 5:** *T*_m_ of PE1 Nanocomposites Obtained by DSC after SSA Procedure^a^

PE1/ZrP-*g*-MPE1_1/5_					
*T*_m_ (°C)	*T*_s_ (°C)				
sample	129.0	124.0	119.0	114.0	109.0
PE1	135.9	124.6	119.2	114.1	109.2
ZrP-*g*-MPE1_1/5_	136.2	126.0	120.9	114.9	109.9
PE1/ZrP-*g*-MPE1_1/5_	135.7	125.1	120.1	114.6	109.6

PE1/ZrP-*g*-MPE2_2/1_					
*T*_m_ (°C)	*T*_s_ (°C)				
sample	128.5	123.5	118.5	113.5	108.5
PE1	135.6	123.8	118.6	113.8	109.8
ZrP-*g*-MPE2_2/1_	136.1	126.0	120.3	114.3	109.1
PE1/ZrP-*g*-MPE2_2/1_	135.9	124.9	119.7	114.3	109.1

PE1/ZrP-*g*-MPE3_2/1_					
*T*_m_ (°C)	*T*_s_ (°C)				
sample	128.5	123.5	118.5	113.5	108.5
PE1	135.6	123.8	118.6	113.8	109.8
ZrP-*g*-MPE3_2/1_	131.9	125.7	119.9	114.4	109.3
PE1/ZrP-*g*-MPE3_2/1_	137.5 and 132.6	125.1	119.7	114.1	109.0

a First five fractionation temperatures and their corresponding *T*_m_ are shown.

On the other hand, separated melting would be expected if phase separation or only partial cocrystallization occurs.^18^ The polar maleated functional group or branching will disrupt PE crystallization, which leads to nonuniform lamellar thickness and multiple melting peaks. SSA final melting profiles of PE1, ZrP-*g*-MPE2_2/1_, and PE1/ZrP-*g*-MPE2_2/1_ are shown in Figure 4b. The main melting peak of PE1/ZrP-*g*-MPE2_2/1_ overlaps with both ZrP-*g*-MPE2_2/1_ and PE1, which indicates that cocrystallization occurs between grafted MPE2 and PE1. However, the melting peak intensities of ZrP-*g*-MPE2_2/1_ at lower temperatures are retained in PE1/ZrP-*g*-MPE2_2/1_, which represents the self-crystallization of grafted MPE2. As a result, only partial cocrystallization is observed and does not stabilize ZrP dispersion in PE1 (Figure 3b). Phase separation was also observed in PE1/ZrP-*g*-MPE3_2/1_ (Figure 4c). The main melting peak splits into two melting temperatures of 137.5 and 132.6 °C, which correspond to PE1 and ZrP-*g*-MPE3_2/1_. The other melting peaks of PE1/ZrP-*g*-MPE3_2/1_ correspond to the thermal fractionation located at the same melting temperature of ZrP-*g*-MPE3_2/1_. The phase separation results in a weak interface and aggregation of ZrP-*g*-MPE3 in PE1.

**Figure 5:**
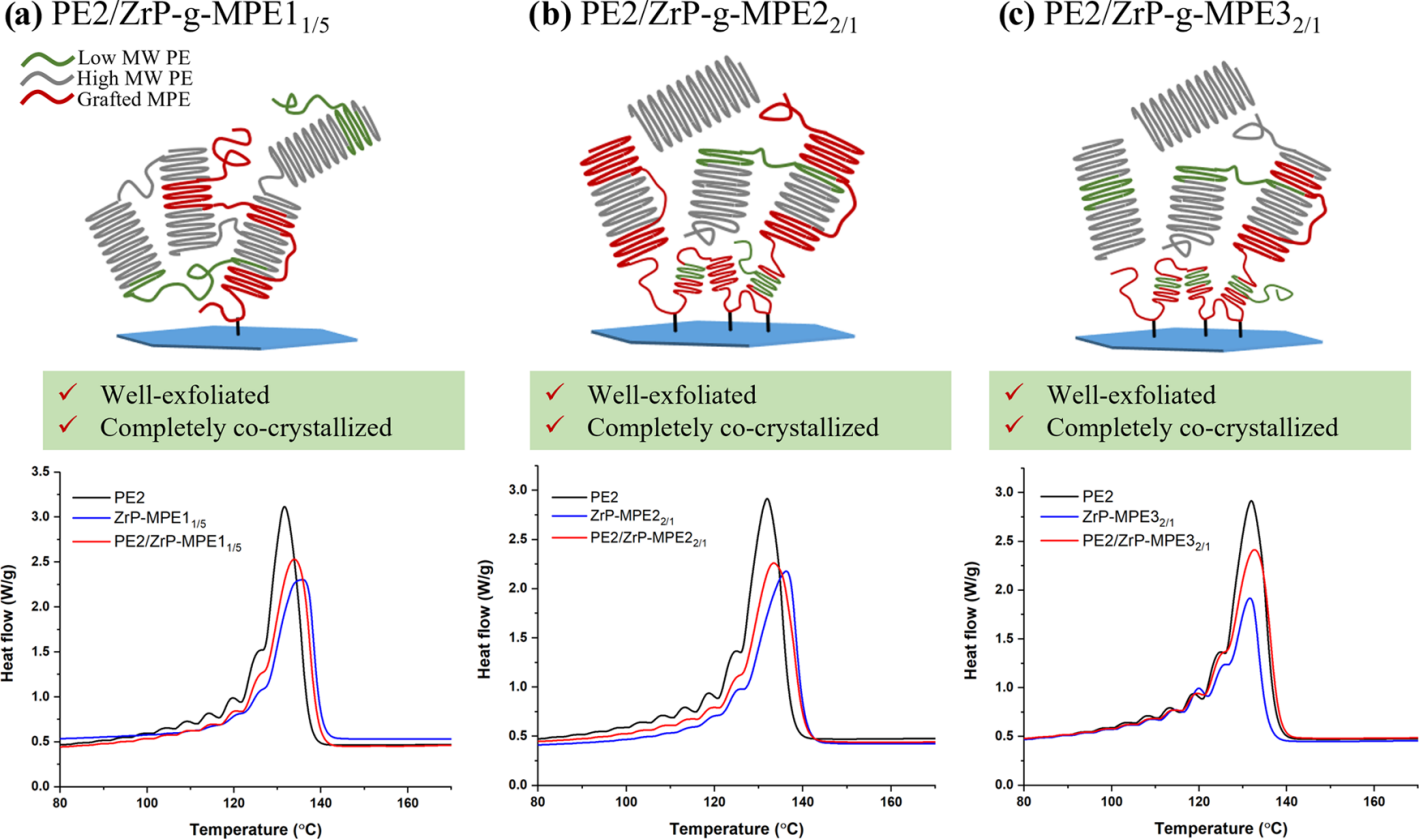
SSA profiles of (a) PE2, ZrP-*g*-MPE1_1/5_ and PE2/ZrP-*g*-MPE1_1/5_, (b) PE2, ZrP-*g*-MPE2_2/1_ and PE2/ZrP-*g*-MPE2_2/1_, and (c) PE2, ZrP-*g*-MPE3_2/1_ and PE2/ZrP-*g*-MPE3_2/1_.

In contrast to PE1, all of the model ZrP-*g*-MPE systems with appropriate MPE graft density can be well dispersed in PE2, which possesses a broader molecular weight distribution and lower crystallinity (Figures 1 and 3). The SSA results of PE2/ZrP-*g*-MPE model systems are shown in Figure 5. The melting temperatures corresponding to the thermal fractionation are summarized in Table 6. All of the PE2/ZrP-*g*-MPE model systems show a similar melting behavior as individual ZrP-*g*-MPE and PE2. In each system, PE2/ZrP-*g*-MPE melting temperatures are located in between those of PE2 and ZrP-*g*-MPE, indicating a complete cocrystallization, which induces a stronger interface. The complete cocrystallization leads to good dispersion of ZrP in PE2.

**Table 6:** *T*_m_ of PE2 Nanocomposites Obtained by DSC after the SSA Procedure^a^

PE2/ZrP-*g*-MPE1_1/5_					
*T*_m_ (°C)	*T*_s_ (°C)				
sample	129.0	124.0	119.0	114.0	109.0
PE2	131.5	125.5	119.5	114.1	109.1
ZrP-*g*-MPE1_1/5_	135.5	126.2	120.6	114.9	109.8
PE2/ZrP-*g*-MPE1_1/5_	133.8	126.0	120.2	114.5	109.6

PE2/ZrP-*g*-MPE2_2/1_					
*T*_m_ (°C)	*T*_s_ (°C)				
sample	128.0	123.0	118.0	113.0	108.0
PE2	131.8	124.8	118.4	113.3	108.3
ZrP-*g*-MPE2_2/1_	136.1	125.5	120.0	114.4	109.1
PE2/ZrP-*g*-MPE2_2/1_	133.5	125.1	119.3	114.0	108.9

PE2/ZrP-*g*-MPE3_2/1_					
*T*_m_ (°C)	*T*_s_ (°C)				
sample	128.0	123.0	118.0	113.0	108.0
PE2	131.8	124.8	118.4	113.3	108.3
ZrP-*g*-MPE3_2/1_	131.4	125.5	119.9	114.1	108.6
PE2/ZrP-*g*-MPE3_2/1_	131.6	125.2	119.0	114.1	108.6

a First five fractionation temperatures and their corresponding *T*_m_ are shown.

### 3.3 Rheological Behavior

The MA content plays an important role in controlling the ZrP dispersion in PE. Grafted MPE with a lower MA content and high MW forms a brush-like morphology on the ZrP surface, while grafted MPE with high MA content results in a mushroom-like morphology. The brush-like MPE grafting with a long chain length allows for cocrystallization with matrix PE to stabilize the ZrP dispersion. As a result, PE1/ZrP-*g*-MPE1 was chosen as a model system to investigate how it influences rheological behaviors and tensile properties.

**Figure 6:**
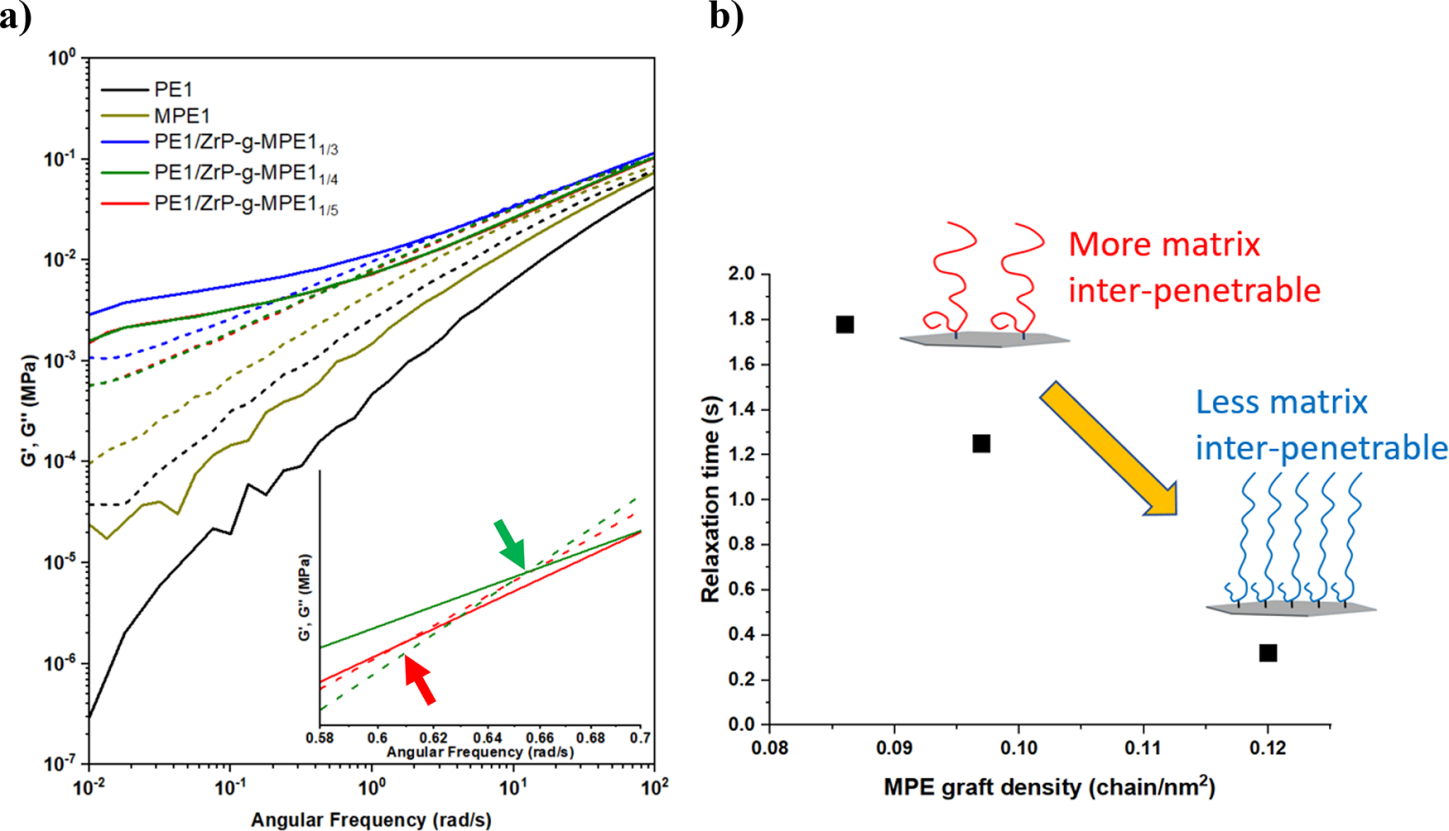
(a) Storage modulus (*G*′) and loss modulus (*G*″) curves of PE1, MPE1, PE1/ZrP-*g*-MPE1_1/5_, PE1/ZrP-*g*-MPE1_1/4_, and PE1/ZrP-*g*-MPE1_1/3_. The solid and dash lines represent the *G*′ and *G*″ values of each system, respectively. The inset plot on the bottom right shows the crossover frequency of PE1/ZrP-*g*-MPE1_1/5_ and PE1/ZrP-*g*-MPE1_1/4_. (b) Entanglement relaxation time vs. MPE graft density.

Rheology is an effective tool for characterizing the interaction between nanoparticles and polymer matrix. To understand the ZrP-*g*-MPE graft density effect on chain entanglement with matrix PE, PE1/ZrP-*g*-MPE1_1/5_, PE1/ZrP-*g*-MPE1_1/4_, and PE1/ZrP-*g*-MPE1_1/3_ were selected as the model systems, which correspond to the MPE1 graft densities of 0.086, 0.097, and 0.120 chain/nm^2^ on the ZrP surface. All ZrP-*g*-MPE1 materials were solution-mixed with PE1 to a ZrP concentration of 4 wt %. ZrP-*g*-MPE dispersed easily in all three systems. The storage (*G*′) and loss modulus (*G*″) results are plotted in Figure 6a. PE containing well-dispersed ZrP-*g*-MPE significantly increases *G*′ and exhibits less frequency dependency when compared to neat PE, especially in the low-frequency region. This indicates the formation of a strong PE–ZrP interface and a pseudo-network formation.^25,26^ The power law relationship is used to fit the curve in the low-frequency region (0.01–0.1 Hz). The **n** values for PE1/ZrP-*g*-MPE1_1/5_, PE1/ZrP-*g*-MPE1_1/4_, and PE1/ZrP-*g*-MPE1_1/3_ are 0.19, 0.25, and 0.26, respectively, which is much lower than that of PE1 (*n* = 1.83). The significantly reduced power law exponent over a wide frequency range indicates the slower and restricted mobility of PE molecules.

The entanglement relaxation time (τ_e_) indicates the time required to achieve configuration rearrangement for the molecular chains. This value can be calculated by the inverse of the crossover frequency of *G*′ and *G*″. With increasing entanglement density, τ_e_ would be prolonged. Here, the τ_e_ values versus MPE1 graft density are plotted in Figure 6b. For MPE1 graft density in the range of 0.087–0.120 chain/nm^2^, the higher MPE1 graft density results in a smaller τ_e_ value. This implies that ZrP-*g*-MPE1_1/5_ is most effective in retarding molecular relaxation by achieving the highest entanglement density at the interface with the PE1 matrix.

### 3.4 Tensile Properties

**Figure 7:**
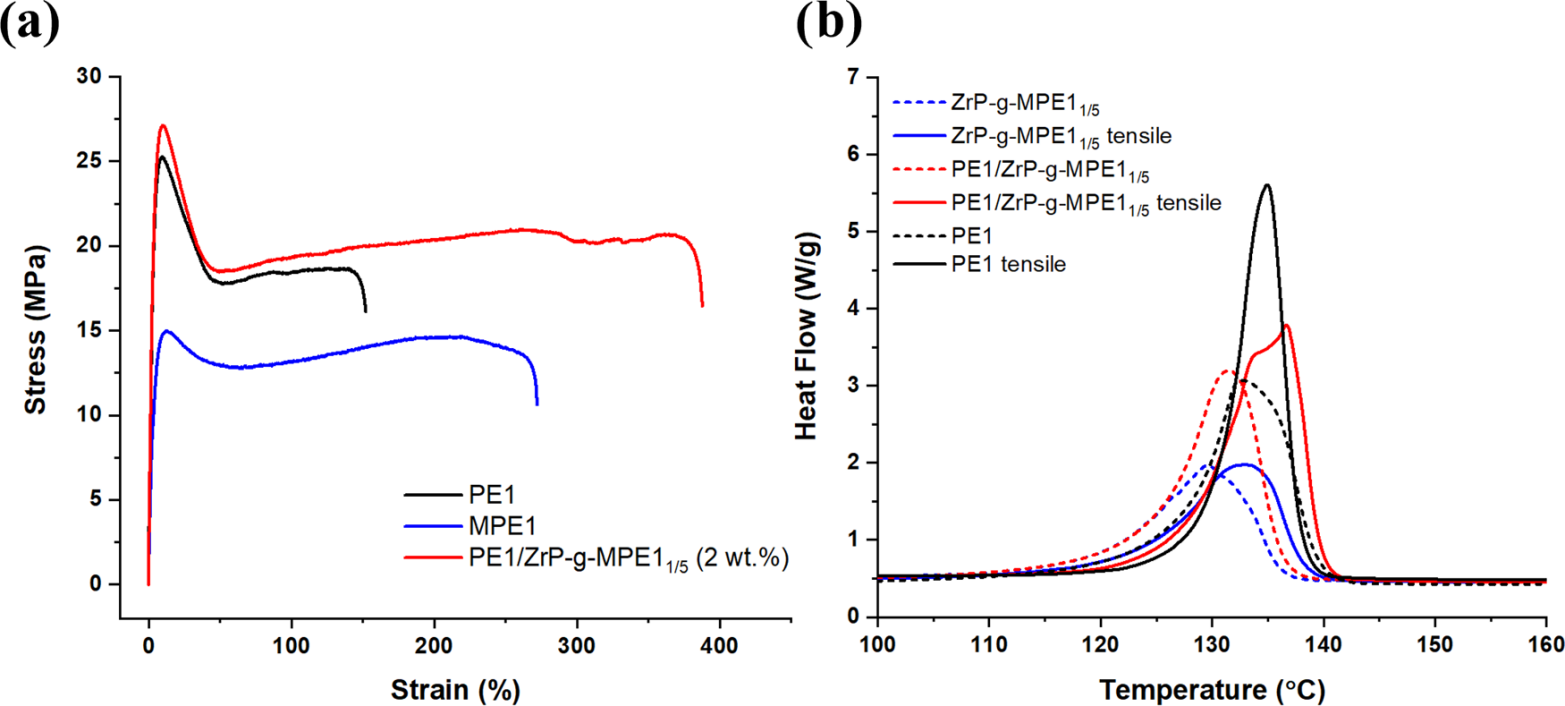
(a) Engineering stress–strain curves of PE1, MPE1, and PE1/ZrP-*g*-MPE1_1/5_ (2 wt % of ZrP). (b) DSC 1st heating scan of the specimens before and after tensile stretching. The dashed lines represent the measurements before the tensile test, and the solid lines represent the measurements after the tensile test (necked region).

**Figure 8:**
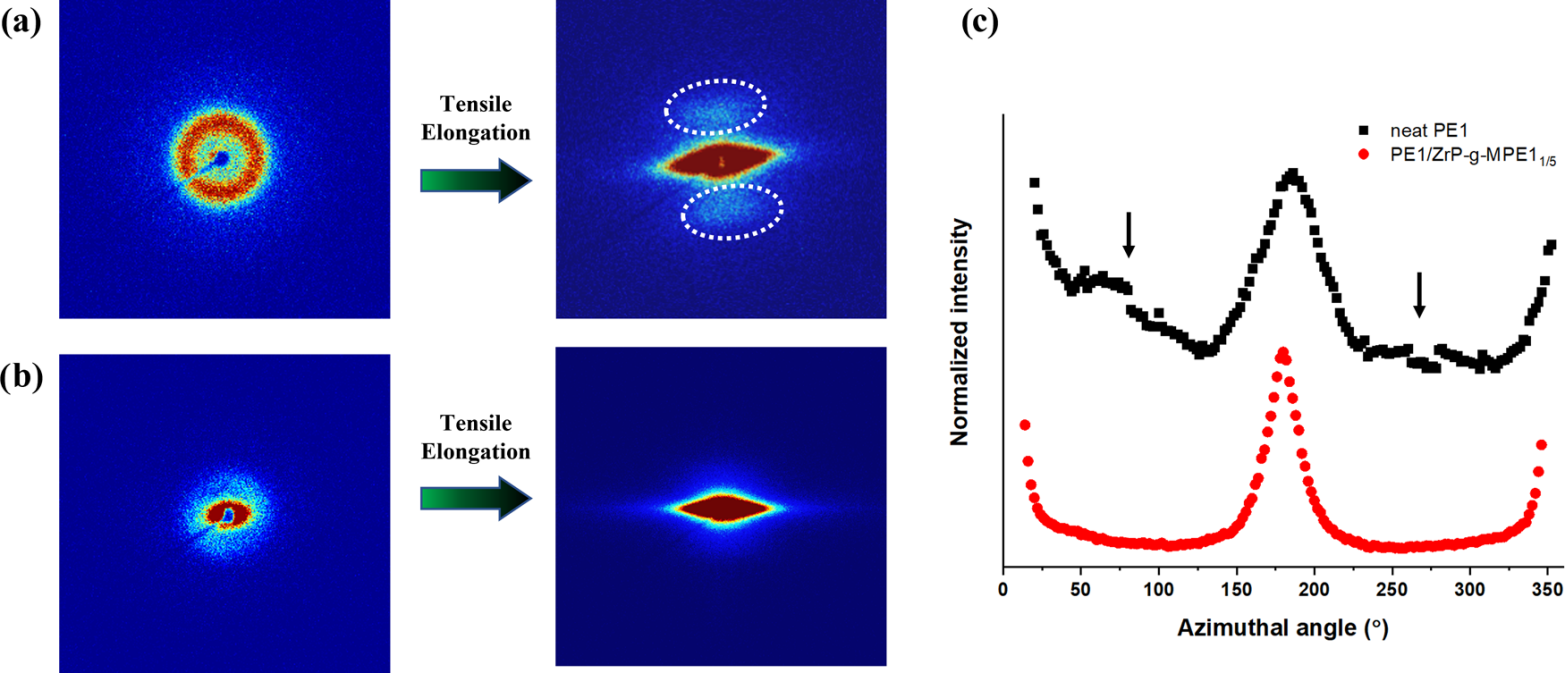
2D SAXS scattering of the necked region of injection molded samples of (a) PE1 and (b) PE1/ZrP-*g*-MPE1_1/5_ (2 wt % ZrP) before and after the tensile test. (c) Azimuthal angle plots of PE1 and PE1/ZrP-*g*-MPE1_1/5_.

Uniaxial tensile tests were performed on the PE1, MPE1, and PE1/ZrP-*g*-MPE1_1/5_ (2 wt %) systems. Their engineering stress–strain curves are shown in Figure 7a. PE nanocomposite crystallinity, Young’s modulus, yield stress, and elongation at break are summarized in Table 7. In PE1/ZrP-*g*-MPE1_1/5_, for which the weight ratio between PE1 and MPE1 is 6.5/1, the presence of MPE1 slightly reduces the nanocomposite crystallinity. Compared to PE1, addition of ZrP (2 wt %) increases the nanocomposite Young’s modulus by 18% and yield stress by 12%. Interestingly, the elongation at break is increased by 150%, which is significantly higher than that of both PE1 and MPE1. To further investigate the nanocomposite deformation mechanism, SAXS and DSC measurements were performed to analyze PE crystal transformation before and after the uniaxial tensile test. DSC 1st heating scan before and after tensile stretching is shown in Figure 7b. SAXS measurements were conducted in the gauge length region before the tensile test and the necked region after the tensile fracture, which are shown in Figure 8.

**Table 7:** Summary of Tensile Properties of PE1, MPE1, and PE Nanocomposites

	crystallinity (%) injection	crystallinity (%) after tensile	Young’s modulus (GPa)	elongation at break (%)	yield stress (MPa)
PE1/ZrP-*g*-MPE1_1/5_ 2 wt %	70	63	0.65 ± 0.034	390 ± 24	28 ± 0.70
PE1	78	70	0.55 ± 0.015	155 ± 63	25 ± 0.86
MPE1	45	40	0.31 ± 0.017	272 ± 25	15 ± 1.12

Tensile stretching has induced PE crystal orientation and increased the lamellar thickness. The calculated lamella thicknesses by SAXS and DSC are summarized in Table S1. The lamellar thickness from SAXS is calculated by *l* = ⌀ × *d*, where *l* is the lamellar thickness, ⌀ is the volume fraction of crystalline PE, and *d* is interlamellar spacing. The lamellar thickness from DSC is calculated by the modified Gibbs–Thomson equation, , where σ is the lamellar surface free energy (5.0 kJ/mol), Δ*h* is the enthalpy of fusion per C_2_-H_4_ unit (8.2 kJ/mol), Δ*z* is the length of the C_2_-H_4_ unit (0.254 nm), and *T*_m_^0^ is the equilibrium melting temperature.^27^ The lamellar thickness calculated from DSC and SAXS are consistent with each other, except for the PE1/ZrP-*g*-MPE1_1/5_ nanocomposite after tensile stretching. In DSC, two separated melting peaks appear. While in SAXS, only one interlamellar d-spacing was captured. The mechanisms of these two techniques are different. DSC characterizes the total amount of heat to melt polymer crystals, while SAXS is based on the average interlamellar d-spacing. This different melting behavior is due to the different PE deformation process in the presence of ZrP-*g*-MPE1_1/5_.

In DSC, two overlapping melting peaks are found. These two broad melting peaks are likely due to two different PE crystal structures near the interface of ZrP-*g*-MPE1_1/5_. After tensile stretching, strain-induced monoclinic PE crystal formation (001 lattice facet) in the system is observed as a broad hump at 2θ = 19.7° via WAXS (Figure S1).^28^ PE deformation mechanisms include interlamellar slip, interlamellar separation, stack rotation, and fine and coarse slip in the crystalline phase.^29,30^ It has been reported that the PE mechanical property is lamellar orientation dependent.^31^ Under tensile deformation, entangled PE chains in the amorphous regions experience chain slip, which leads to the lamellar slip or separation. After yielding, cavitation and PE crystal deformation begin. The crystal deformation reduces PE crystallinity (Table 7). At this stage, a portion of the original PE orthorhombic crystals undergo Martensitic transformation and form monoclinic PE crystals. It has been reported that the monoclinic PE phase may improve ductility and toughness.^32,33^ Finally, at a higher strain, PE chains in the amorphous region are further stretched to eventually form a strain-induced crystalline structure.^29,34^

In the PE1/ZrP-*g*-MPE1_1/5_ system, because of the chain entanglement and complete cocrystallization at the interface, chain slip and formation of monoclinic PE crystals after yielding contribute to the improved ductility. It is important to note that the metastable monoclinic PE crystals usually form upon yielding.^28,32,33^ In Figure 8, PE1 after the tensile test possesses two-point meridional scattering (see the arrows), which indicates that a portion of the crystalline structure was reoriented toward the tensile elongation direction. Only a single strong peak can be observed in PE1/ZrP-*g*-MPE1_1/5_ before and after tensile stretching, which indicates that the lamella of PE1/ZrP-*g*-MPE1_1/5_ was orientated perpendicular to the injection molding direction and not affected by the tensile stretching.^34−36^ The presence of well-dispersed ZrP and strong cocrystallization restrict the rotation of the PE lamella compared to neat PE1.^35^ The formation of the monoclinic PE crystal structure and remnant of the deformed PE crystals result in the observed multiple melting peaks in PE1/ZrP-*g*-MPE1_1/5_ after the tensile test. At present, the physics behind the effect of ZrP on monoclinic PE crystal formation and how it affects PE ductility is still not clear. More work is still needed.

At present, the high energetic and entropic barriers for dispersing 2D nanoplatelets in polyolefin matrices have been overcome by allowing for cocrystallization to take place at their interface. Here, MPE was successfully grafted onto an already exfoliated ZrP surface. By tuning the MA content, molecular weight, and graft density of MPE, a semidilute brush-like grafted MPE structure on the ZrP surface was achieved, which leads to complete cocrystallization with the PE matrix, and stabilizes ZrP dispersion. After achieving the well-designed ZrP-*g*-MPE structure, additional surface functional groups can be immobilized on the nanoparticle surface, which could broaden the multifunctional application of polyolefin nanocomposites, i.e., flame retardancy, recyclability, UV shielding, protein delivery, etc.^37−41^

## 4 Conclusions

The PE–ZrP interface was systematically controlled by tuning the MA content, molecular weight, and graft density of MPE. Grafting MPE with a lower MA content onto the ZrP surface tends to form an extended polymer brush-like morphology, which stabilizes ZrP dispersion by cocrystallization at the interface. MPE with high MA content forms a mushroom-like grafted PE morphology, which induces partial cocrystallization with matrix PE and relatively poor dispersion of ZrP in high crystallinity PE. In the brush-like ZrP-*g*-MPE model system, a lower MPE graft density promotes strong entanglement with matrix PE. With the well-controlled interface between ZrP and PE, PE/ZrP-*g*-MPE nanocomposites exhibit a higher modulus and a more ductile behavior. The present study introduces a robust approach to preparing well-dispersed polyolefin nanocomposites with attractive properties. This approach is suitable for the preparation of different kinds of polymer nanocomposites to achieve a wide range of engineering applications.

## Supporting Information Available

Lamellar thickness calculated by DSC and SAXS; and WAXS of PE1 and PE1/ZrP-*g*-MPE1_1/5_ before and after the tensile test (PDF)
