# Supplementary material for: Preparation of Polyethylene/α-Zirconium Phosphate Nanocomposites via a Well-Controlled Polyethylene-Grafted Interface
Source: Langmuir. 2023 Apr 13;39(16):5803–13. doi: 10.1021/acs.langmuir.3c00058 (PMC10853957; doi:10.1021/acs.langmuir.3c00058)
Supplement: Supplementary file 1 — la3c00058_si_001.pdf [file la3c00058_si_001.pdf]

Preparation of Polyethylene/α-Zirconium Phosphate Nanocomposites via Well-Controlled Polyethylene-Grafted Interface

Mingzhen Zhao[a], Hong-Mao Wu[b], Hengxi Chen[a], Guan-Hui Lai[a], Zewen Zhu[a], Jen-Long Wu[b], Wen-Hao Kang[b] and Hung-Jue Sue[a,*]

[a]: *Department of Material Science and Engineering, Texas A&M University, College Station, TX, 77843, USA*
[b]: *Polyolefin department of Formosa Plastics Corporation, Yunlin County, 63801, Taiwan*

Table S1. Lamellar thickness calculated by DSC and SAXS.

Techniques \ Lamella thickness (nm)	Injection molded			After tensile stretching		
	ZrP-MPE1$_{1/5}$	PE1	PE1/ZrP-MPE1$_{1/5}$	ZrP-MPE1$_{1/5}$	PE1	PE1/ZrP-MPE1$_{1/5}$
SAXS	10.89	13.92	13.39	18.19	21.40	24.61
DSC	10.72	14.56	12.87	15.85	19.45	16.90 and 26.31

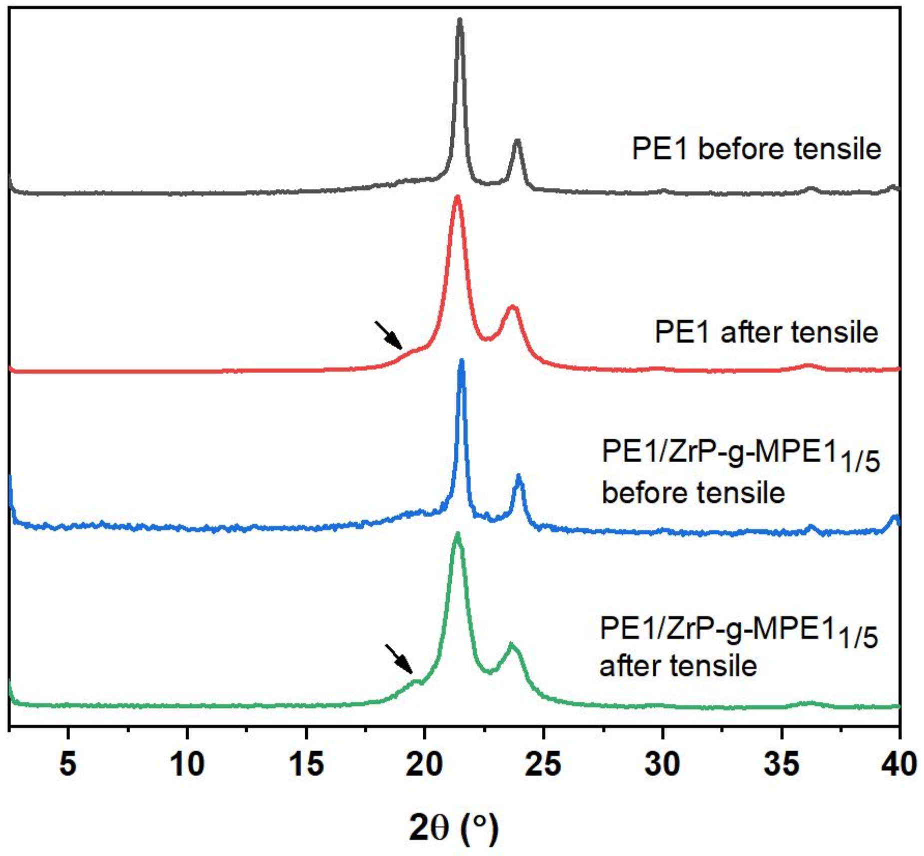

Figure S1. WAXS of PE1 and PE1/ZrP-g-MPE1$_{1/5}$ before and after tensile test. All the tested specimens were cut in the necked region. The arrows shown in the plot represent the monoclinic PE lattice structure.
